# Evaluation of potential drug- drug interactions among Palestinian hemodialysis patients

**DOI:** 10.1186/s12882-016-0317-4

**Published:** 2016-07-26

**Authors:** Rowa’ Al-Ramahi, Afnan R. Raddad, Alaa O. Rashed, Amneh Bsharat, Dania Abu-Ghazaleh, Eman Yasin, Oraina Shehab

**Affiliations:** Department of Pharmacy, Faculty of Medicine and Health Sciences, An-Najah National University, Nablus, Palestine

**Keywords:** Drug-drug interactions, Hemodialysis, Palestine

## Abstract

**Background:**

The aims of this study are to find the prevalence of potential drug-drug interactions (DDIs) in patients with Hemodialysis and identify factors associated with these interactions if present.

**Methods:**

The study was an observational- retrospective cohort study that was conducted in ten hemodialysis units in the West bank, Palestine, between June and August 2015. The data collection form was completed by interviewing the patients in addition to reviewing their medical records. Potential DDIs were reviewed. SPSS program was used for data analysis.

**Results:**

The study included 275 patients, a total of 930 potential interactions were identified in 245 (89.1 %) patients. Patients were prescribed 1–15 drugs with a mean (± SD) of 7.87 ± 2.44, calcium carbonate was the most common drug prescribed. The most common potential interaction in 114 (41.5 %) patients was Calcium Carbonate/Amlodipine followed by Calcium Carbonate/Aspirin in 76 (27.6 %) cases. Most patients (89.9 %) of the patients had one or more comorbid diseases; hypertension, diabetes and gout were the most common. Univariate analysis showed that the number of potential DDIs were related to the number of diseases, the number of prescribed drugs (*P* value <0.0001) and the age of the patient (*P* value = 0.015). The results of multiple linear regression showed a significant positive association between number of potential DDIs with the total number of medications (*r* = 0.242; *p* < 0.001).

**Conclusion:**

The prevalence of potential drug- drug interactions among hemodialysis patients is very common; they are highly expected and depend on the number of drugs taken by the patients. Many of these potential interactions are considered as preventable drug- related problems, so screening for potential interactions and monitoring regularly is highly needed.

## Background

Chronic Kidney Disease (CKD) is a global public health concern [1]. End-stage renal disease (ESRD) and CKD are increasing worldwide [2, 3]. The variation among communities in the incidence of ESRD mirrors that in the prevalence of diabetes, obesity, and hypertension [4]. The prevalence of ESRD patients on dialysis in the West Bank, Palestine is comparable with that of adjacent countries, but is still far below the high prevalence rate recorded in Western countries [5].

Patients who go through ESRD and who receive hemodialysis (HD) are expected to be given 10 to 12 medications daily and many of these medications require multiple doses each day. Due to poly-pharmacy, frequent medication adjustments on dialysis versus non- dialysis days, medically unstable nature of the disease and restricted life styles, these patients are at high risk for developing drug related problems (DRPs) and non-adherence. With such a large number of medications, there is an increased risk for drug-drug interactions (DDIs) [6], which is associated with significant morbidity, impaired quality of life, mortality, and others such as primary drivers of hospital admissions and health care costs. The specialist must keep in mind that pharmaceutical options must be balanced with the potential risk of multiple medication use. The potential risks include, but are not limiting to, adverse effects, inappropriate dosing regimens, drug-disease interactions and drug-drug interactions [7].

Even though, DDIs are considered as preventable medication related problems, studies revealed that up to 11 % of patients experience symptoms associated with DDIs and these are responsible for approximately 2.8 % of hospital admissions [8]. It is important to remember that recognizing a potential DDI in a patient, does not mean he will suffer from an actual DDI and a clinically significant effect, sometimes extra monitoring is the only action needed. Monitoring of potential DDIs may improve the quality of prescribing and dispensing and it might form a basis for education focused on appropriate prescribing [9].

It is hoped that this study will help raise awareness of the importance of drug monitoring and review in hemodialysis patients. Help both clinicians and researchers to have a better view on this topic in terms of prevalence and risk factors and guide clinicians on how to avoid such interactions. If such interactions can be avoided this will increase the patients’ quality of life, survival and satisfaction.

The aims of this study are to find the prevalence and nature of potential drug-drug interactions (DDIs) in patients with Hemodialysis and identify factors associated with these interactions if present.

## Methods

### Study design

An observational- retrospective cohort study was carried out; it included ESRD patients undergoing hemodialysis in ten hemodialysis units in the West bank, Palestine, between June and August 2015. The data collection form was completed by interviewing the patients in addition to reviewing their medical records. All hemodialysis patients who visited the hemodialysis centers in the included hospitals during the study period were asked to participate in this study if they meet the inclusion criteria. The inclusion criteria were as follows: all adult patients (≥18 years) who were diagnosed as ESRD and were on maintenance dialysis.

The study protocol was approved by local institutional review boards (IRB) of An -Najah National University and the Ministry of Health before the beginning of this study. All subjects had been informed of their rights to refuse or discontinue participation in the study according to the ethical standards. Informed verbal consent from each eligible patient was obtained before beginning the interviews.

### Sample size

According to the Ministry of Health records of 2013 in Palestine, the total number of hemodialysis Patients across ten dialysis units in the West Bank was 800 [10]. An automated software program, (Raosoft sample size calculator: (http://www.raosoft.com/samplesize.html) was used to calculate the required sample size for this study and it was 260 patients. In order to minimize erroneous results and increase the study reliability, the target sample size was increased 5 to 10 %. All of the patients who were asked to participate in this study accepted to be involved.

### Data collection

Patient’s medical records were reviewed and a questionnaire was used. Patient’s age, gender, medical conditions, all prescribed medications and dosage regimens were documented from the patient’s charts. The questionnaire was used to confirm the data obtained from the records and collect additional data from the patients regarding sociodemographic characteristics, other medical conditions, prescribed and non- prescribed medications and herbs. All of the information was tabulated for analysis. A software program for drug interactions by LexiComp was used for screening the potential DDIs. According to this system each interaction was assigned a risk rating of A, B, C, D, or X. Risk rating A means no known interaction, for B no action is needed, C requires to monitor therapy, D requires to consider therapy modification, while X means we should avoid combination.

### Statistical analysis

Statistical analysis was performed using Statistical Package for Social Sciences (SPSS version 16.0). Mean ± standard deviation was computed for continuous data. Frequencies and percentages were calculated for categorical variables. Kolmogorov-Smirnov test was used to evaluate the normal distribution of the variables and Mann-Whitney test or Kruscal Wallis test was used according to that. Multiple linear regression was performed also. Probability (p) value of less than 0.05 was considered to be statistically significant for all analyses.

## Results

### Socio-demographic and clinical characteristics of the patients

During the study period, a total of 275 patients were interviewed. Among the surveyed patients, 151 (54.9 %) were males. The average age (± SD) of patients was 50.67 ± 15.93 years, and the maximum age was 82 years. Most of the patients 263 (95.6 %) lived with their families. The majority of patients 189 (68.7 %) were living in a village. Regarding their educational level, a high percentage of patients were illiterate or had primary school only 114 (41.5 %). Most patients 231 (84.0 %) patients were unemployed, 217 (78.9 %) patients were smokers and 220 (80.0 %) were married.

### Dialysis data

In this study there was one patient who spent 25 years on HD. One month was the minimum duration, and 300 months was the maximum. The length of each dialysis session was 210 min in most patients (199; 72.4 %), other options were 120 in 1 case (0.4 %), 180 in 2 cases (0.7 %) or 240 in 73 cases (26.5 %). Most patients (218; 79.3 %) had three hemodialysis sessions per week, 54 patients (19.6 %) were on two sessions per week, 2 patients (0.7 %) were on 4 sessions per week and one patient (0.4 %) was on one session per week.

### ESRD causes and co-morbid conditions

Many causes can lead to the ESRD, for which HD should be initiated. A high number of HD patients in this study had hypertension as cause of ESRD; 85 (30.9 %), followed by diabetes mellitus in 81 (29.5 %) cases, followed by unknown causes in 54 (19.6 %) cases, then polycystic kidney disease in 17 (6.2 %) cases.

Hypertension, diabetes mellitus, gout, myocardial infarction, hyperlipidemia, and congestive heart failure respectively, were the most commonly co-morbid conditions present in the HD patients, with different combination between these conditions in each patient. Among patients, 216 (78.5 %) had hypertension, 117 (42.5 %) had diabetes mellitus (type 1 & 2), 26 (9.5 %) had gout, 23 (8.4 %) had myocardial infarction, 17 (6.2 %) had hyperlipidemia, and 16 (5.8 %) had congestive heart failure.

### Prescribing pattern of medications in HD patients

During the study period, a total of 90 different medications were used by the patients. The patients were taking a minimum of 1 and a maximum of 15 medications, with a mean (± SD) of 7.87 ± 2.44. The most commonly prescribed medications were calcium carbonate being used by 212 (77.1 %). Followed by alfacalcidol, iron/folic acid, aspirin and amlodipine which were used by 203 (73.8 %), 180 (65.5 %), 151 (54.9 %), 136 (49.5 %) patients respectively.

### Evaluation of potential drug-drug interactions (DDIs)

**Fig. 1:**
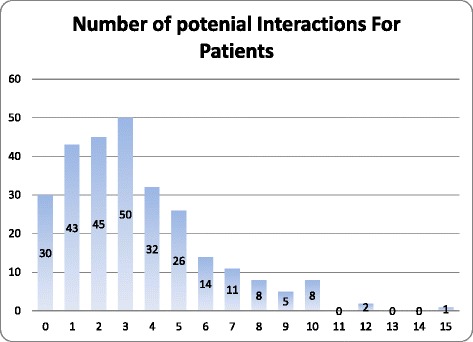
The frequency of the number of Potential drug-drug interactions

Among 275 patients, 245 (89.1 %) patients had at least one potential DDI (Fig. 1). Among the 245 patients who had potential interactions, a total of 930 interactions were identified. According to risk rating classification, 676 (72.69 %) were C, 172 (18.49 %) were B, 78 (8.39 %) were D, and 2 (0.22 %) for each A and X risk rating.

The most common interaction in 114 (41.5 %) patients was calcium carbonate with amlodipine (Calcium salts may diminish the therapeutic effect of Calcium Channel Blockers) followed by aspirin with calcium carbonate in 76 (27.6 %) cases (Antacids may decrease the serum concentration of Salicylates). Table 1 shows the top ten potential interactions.

**Table 1:** Top Ten potential drug-drug interactions

NO.	Drug-Drug interaction	Frequency	Risk rating
1.	Calcium Carbonate/Amlodipine	114	C
2.	Calcium Carbonate/Aspirin	76	B
3.	Aspirin/Furosemide	74	C
4.	Aspirin/Enoxaparin	40	C
5.	Aspirin/Insulin	39	C
6.	Calcium Carbonate/Ranitidine	33	B
7.	Calcium Carbonate/Atenolol	25	B
8.	Amlodipine/Atenolol	21	C
9.	Amlodipine/Enalapril	20	C
10.	Furosemide/Atenolol	19	C

### Factors associated with potential drug-drug interactions

Univariate analysis showed a significant correlation between age and number of potential interactions (*p* value = 0.015). The number of potential DDIs that the patients had were also related to the number of chronic diseases and total number of medications (*P* value <0.001 for each one). However, there was no significant relationship with gender (*p* value =0.725), smoking status (*p* value =0.834) and the duration of dialysis (*p* value = 0.920) (Table 2). The results of multiple linear regression showed a significant positive association between number of potential DDIs with the total number of medications (*r* = 0.242; *p* <0.001). The range of VIF was from 1.067 to 1.206 which indicated absence of multicollinearity between independent variables (Table 3).

**Table 2:** Univariate analysis of factors associated with potential drug-drug interactions

Characteristic	Frequency % *N* = 275	Number of DDIs Median (Q1-Q3)	*P*-value
Gender			
Male	151 (54.9)	3 (1–5)	0.725^a^
Female	124 (45.1)	3 (1.25–5)	
Age category			
<30	40 (14.5)	2 (1–3)	0.015^b^
30–60	151 (54.9)	3 (1–5)	
>60	84 (30.5)	3 (2–5)	
Smoker			
Yes	58 (21.1)	3 (1–5)	0.834^a^
No	217 (78.9)	3 (1–5)	
Duration of dialysis (years)			
<4	203 (73.8)	3 (1–5)	0.920^a^
≥4	72 (26.2)	3 (2–4)	
Total chronic co-morbid disease			
None	36 (13.1)	1 (1–3)	<0.001^b^
1	96 (34.9)	2.5 (1–4)	
2	90 (32.7)	3 (1.75–5)	
≥3	53 (19.3)	4 (2–7)	
Total number of medications			
<5	26 (9.5)	1 (0.0–1)	<0.001^a^
≥5	249 (90.5)	3 (1–5)	

^a^Mann- Whitney test

^b^Kruscal Wallis test

**Table 3:** Multivariate analysis of factors associated with potential drug-drug interactions

Characteristic	Standardized coefficients	*P*-value	95 % Confidence Interval for B	
	Beta		Lower bound	Lower bound
Number of medications for each patient	0.556	<0.001	0.196	0.288
Patient age group	0.045	0.381	−0.090	0.235
Number of comorbid diseases the patient have	0.049	0.367	−0.056	0.151

## Discussion

The patients in this study had many comorbidities. Hypertension, diabetes mellitus, gout, and myocardial infarction were the most common co-morbid conditions present in the HD patients respectively. Regarding the cause, there are many causes that can lead to the ESRD, for which HD should be initiated. A high number of HD patients in this study had hypertension as a cause of ESRD, followed by diabetes mellitus and polycystic kidney disease. In comparison with other studies, in a study from Thailand and another from Latin America, the most common cause of ESRD was diabetes mellitus [11, 12].

In this study, the 275 HD patients were prescribed 90 different drugs with a mean (± SD) of 7.87 ± 2.44. The number of medications in this study is close to a Japanese study where the mean was around 7.2 medications [13]. However, this result is lower than a study in Malaysia that included ESRD patients where the mean was around 9 medications [14]. Also, this is lower than a study from the USA where the mean of medications prescribed for HD patient reached 12.3 ± 0.5 medications [15]. In summary, polypharmacy is very common in ESRD patients.

The most commonly prescribed medications in current study were calcium carbonate, followed by alfacalcidol, iron/folic acid, aspirin and amlodipine. When compared with a study conducted at a nephrology unit in a Malaysian hospital, the top five used medications were calcium carbonate (which was the most commonly prescribed medication also), followed by a combination of folic acid and vitamin B complex, and the third commonly prescribed medication was metoprolol followed by lovastatin and ferrous sulfate [14]. Another cross-sectional, observational study was conducted in nephrology department of a government hospital in India, the five most commonly prescribed drugs were multivitamins, iron, folic acid, calcium carbonate, and calcitriol [16]. Due to this poly-pharmacy, HD patients are at higher risk for drug related problems including DDIs.

In the present study, we found that the prevalence of potential DDIs in medications of CKD patients was 89.1 %, this is a very high percentage. Another high prevalence was seen in a study from the India where the potential DDIs among renal failure patients reached 76.09 % [6]. When actual DDIs were evaluated in a study performed in U.S by Grabe et al., they accounted for 27.5 % of detected drug related problems [17].

There are different methods that can be used to identify and classify drug-drug interactions that have been used in different studies. In current study, LexiComp® was used for this purpose. Most of the potential DDIs were C-risk rating which means that monitoring is needed. In 78 potential DDIs detected, therapy modification was required according to the recommendations as their risk rating was D. In a prospective, observational study that was carried out in a South Indian tertiary care hospital, every combination of prescribed drug was analyzed using the Thomson Reuters Micromedex® DrugReax® system to look for potential DDIs, among the 156 patients, 474 potential drug interactions were identified. Most of them were delayed in onset, moderate in severity, pharmacodynamic in nature, and belong to level 2 i.e., moderate in significance [6]. This confirms that not in all cases of potential DDIs we need to stop the combination.

Univariate analysis showed that potential DDIs were affected by age, number of diseases and drugs. This is similar to a study from the USA which showed that older age and number of drugs prescribed were more likely to lead to major interactions [18]. It is recommended that more attention should be given to patients with chronic diseases as hypertension and diabetes, which are considered the most common co-morbidity among our HD patients, and that all their drugs should be documented and screened for potential DDIs.

Among different categories of drugs involved in potential DDIs, most of them were those of daily use to treat patients with chronic disorders. The most common potential interaction was calcium carbonate with amlodipine, followed by aspirin with calcium carbonate and then aspirin with furosemide, enoxaparin, and insulin. However, in another study performed by Rama et al. (2012), they found that beta blockers, calcium channel blockers, diuretics, digoxin and antiarrhythmic agents, were the most common medication involved in the list of potential DDIs. The most frequently prescribed drug combinations with potential DDI were ascorbicacid/cyanocobalamine, clonidine/metoprolol, amlodipine/metoprolol and insulin/metoprolol [6].

Physicians and pharmacists should be more aware of these potential interactions and they should collaborate to develop educational programs and improve patients’ counseling to avoid improper use of medications. Further researches and actual steps are required to optimize patient outcomes and minimize the prevalence of these interactions. It is important to remember that not all DDIs are harmful. Some of these combinations can be beneficial. Our recommendation is to give more attention for patient’s medication list before considering this combination as desirable or undesirable drug interaction.

The first limitation of this study is that the sampling method was convenient, that limits the ability to make broader generalizations from the results. Another limitation is that the honesty of patient to report non -prescribed drugs and other information was no guaranteed. However, this study is the first of its type in Palestine. These results can give a baseline data that can be useful in finding the prevalence of potential drug-drug interactions in patients with ESRD and identify factors associated with these interactions if present and in designing and implementing suitable interventions, educational programs and performing other related studies.

## Conclusion

The prevalence of potential drug- drug interactions among hemodialysis patients is very common; they are highly expected and depend on the number of drugs taken by the patients which were used to treat co-morbid conditions. Many of these interactions are considered as preventable medication related problems, so screening for potential interactions and monitoring regularly should take place routinely before prescribing any medication to improve quality of prescribing and dispending.

## Abbreviations

CKD, Chronic Kidney Disease; DDIs, Drug-Drug Interactions; DRPs, Drug Related Problems; ESRD, End-Stage Renal Disease; HD, Hemodialysis
